# Targeting Peptidylarginine Deiminase 3 to Efficiently Suppress Herpes Simplex Virus Type 2 Infection

**DOI:** 10.3390/ijms25168709

**Published:** 2024-08-09

**Authors:** Selina Pasquero, Francesca Gugliesi, Matteo Biolatti, Camilla Albano, Greta Bajetto, Linda Trifirò, Stefano Raviola, Valentina Dell’Oste, Marco De Andrea

**Affiliations:** 1 Department of Public Health and Pediatric Sciences, Medical School, University of Turin, 10124 Turin, Italy; selina.pasquero@unito.it (S.P.);; 2 CAAD Center for Translational Research on Autoimmune and Allergic Disease, University of Piemonte Orientale, Novara Medical School, 28100 Novara, Italy; 3 Department of Translational Medicine, University of Piemonte Orientale, 28100 Novara, Italy

**Keywords:** citrullination, protein arginine deiminase type 3 (PAD3, PADI3), herpes simplex virus type 2 (HSV-2), host-targeting antivirals (HTAs)

## Abstract

Protein expression is regulated through multiple mechanisms, including post-translational modifications (PTMs), which can alter protein structure, stability, localization, and function. Among these, citrullination stands out due to its ability to convert arginine residues into citrulline, altering protein charge and mass. This modification is catalyzed by calcium-dependent protein arginine deiminases (PADs), enzymes implicated in various inflammatory diseases. We have recently shown that human cytomegalovirus (HCMV) and herpes simplex virus type 1 (HSV-1) exploit these enzymes to enhance their replication capabilities. Although the role of PADs in HCMV and HSV-1 infections is well documented, their involvement in HSV-2 infection has not yet been thoroughly investigated. Here, we demonstrate that HSV-2 manipulates the overall protein citrullination profile by activating three PAD isoforms: PAD2, PAD3, and PAD4. However, as previously observed during HSV-1 infection, PAD3 is the most significantly upregulated isoform, both at the mRNA and protein levels. Consistently, we demonstrate that inhibiting PAD3, either through the specific inhibitor CAY10727 or via CRISPR/Cas9-mediated gene silencing, markedly reduces HSV-2 replication and viral protein expression. Lastly, we show that CAY10727 displays an IC50 value of 0.3 μM, which is extremely close to what was previously observed for HSV-1. Overall, our findings highlight the crucial role of PAD3 in the life cycle of HSV-2 and suggest that the targeted inhibition of PAD3 may represent a promising approach for treating HSV-2 infections, especially in cases resistant to existing antiviral therapies.

## 1. Introduction

Protein expression is regulated in multiple stages to ensure the appropriate synthesis of these molecules. Following translation, proteins experience chemical modifications via enzymatic and nonenzymatic reactions. These post-translational modifications (PTMs) can alter protein structure, stability, sub-cellular positioning, and functionality and can regulate their interactions with other binding partners. Importantly, viruses exploit PTMs to control the activity of key host proteins [1,2].

Over 200 types of PTMs have been described to date [3], including specific amino acid side-chain modifications and peptide bond cleavage. Among these, protein citrullination, also defined as deimination, involves the enzymatic conversion of the imine group of a peptidyl-arginine residue into a carbonyl group, thereby forming the amino acid citrulline, a non-genetically encoded amino acid with a mass increase of 0.984 Da and the loss of one positive charge [4,5].

Citrullination is driven by the calcium-dependent protein arginine deiminase (PAD) family of enzymes [6], which in mammals includes five isoforms (PADs 1–4 and 6). Each isoform exhibits specific expression patterns in different tissues and substrate preferences. The overexpression of PAD enzymes has been observed in various inflammatory conditions, implying that abnormal citrullination could play a pathogenic role in diseases associated with inflammation [7,8,9,10]. Table 1 summarizes detailed information on PAD expression, biological activity, and disease correlation.

In recent years, a relationship between citrullination and viral infections has been established [16,17,18,19]. For instance, LL37 peptide antiviral activity seems to be hindered following human rhinovirus (HRV)-induced citrullination [16], and PAD4 is significantly increased in HCoV-OC43-infected MRC-5 cells, where its inhibition strongly affects viral replication [18]. Moreover, artificially citrullinated Epstein–Barr virus (EBV) proteins were specifically recognized by sera from RA patients [17], and total protein citrullination and PAD4 expression levels were associated with HPV-induced cervical cancer progression [19]. Consistently, our group has demonstrated that human cytomegalovirus (HCMV) induces PAD2- and PAD4-mediated citrullination, and that inhibiting this process with the pan-PAD inhibitor Cl-amidine (Cl-A) impairs viral replication [20]. More recently, we have shown that an additional member of the *Herpesviridae* family, herpes simplex virus 1 (HSV-1), also affects the protein citrullination profile during infection. Unlike HCMV, HSV-1 infection specifically induces PAD3 expression, and depletion of this isozyme by specific PAD3 inhibitors or siRNAs strongly dampens HSV-1 replication [21]. These studies indicate that herpesviruses have evolved multiple strategies to manipulate the expression of distinct PAD family members to sustain their replication. However, the role of PADs and protein citrullination during herpes simplex virus 2 (HSV-2) infection remains unexplored.

HSV-2 is a highly prevalent sexually transmitted infection, affecting millions of individuals worldwide. Beyond the discomfort of recurrent genital lesions, HSV-2 infections can lead to severe complications, including neonatal herpes and an increased risk of acquiring or transmitting HIV [22]. Classical clinical manifestations include painful lesions characterized by vesicular lesions clustered on the skin and genital mucous membranes. In immunocompetent individuals, these lesions regress spontaneously, but the virus remains dormant in the periaxonal sheath of sensory nerves, with possible sporadic reactivations throughout the individual’s life [23]. The primary infection is usually acquired sexually in adulthood. However, vertical transmission from mother to child at birth can occur, leading to serious consequences for the newborn, such as disseminated infection, encephalitis, and neurological complications [24].

Despite the availability of antiviral drugs that target DNA polymerase, challenges such as the emergence of drug-resistant strains and limitations in efficacy and tolerability persist [25]. Therefore, the development of novel antiviral agents targeting HSV-2 is critical to expand treatment options, improve patient outcomes, and eventually reduce the burden of HSV-2-related morbidity and transmission.

Against this backdrop, host-targeting antivirals (HTAs) represent a novel strategy for developing antiviral drugs that can overcome these challenges by focusing on host–cell components implicated in viral replication [26,27].

In the present study, we examine the interaction of HSV-2 with the host cellular machinery to define the functional role of PADs and citrullination during this type of viral infection. Furthermore, we investigate the antiviral activity of different PAD inhibitors against HSV-2. We show that HSV-2 infection modifies the overall citrullination profile in human foreskin fibroblasts (HFFs). Similar to HSV-1, HSV-2 achieves this by mainly manipulating the expression of PAD3 at both the mRNA and protein levels. Consistently, we show that inhibiting PAD3 through the specific pharmacological inhibitor CAY10727, or via gene silencing using the CRISPR/Cas9 technique, significantly impairs HSV-2 replication.

## 2. Results

### 2.1. HSV-2 Infection Induces Protein Citrullination through PAD Upregulation

In our previous works, we have shown that members of the Herpesviridae family, such as HCMV and HSV-1, trigger PAD-mediated citrullination to boost their replication. To determine whether this phenomenon also applies to HSV-2, we first assessed total protein citrullination in HFFs after HSV-2 infection (multiplicity of infection, MOI = 1) at different time points post infection.

**Figure 1:**
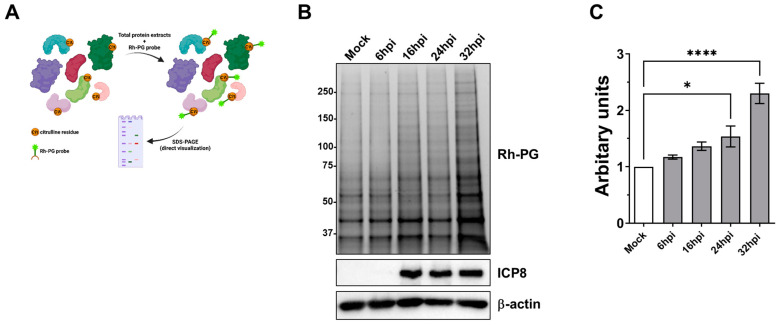
HSV-2 infection affects the protein citrullination pattern in HFFs. (**A**) Schematic representation of the method used for labeling citrullinated proteins in cellular protein lysates using the Rh-PG probe. Created with BioRender.com. (**B**) Protein lysates from mock-infected or HSV-2-infected HFFs, collected at different hpi, were treated with the Rh-PG probe and analyzed through gel electrophoresis to identify citrullinated proteins. An anti-ICP8 antibody confirmed HSV-2 infection, while β-actin cellular expression served as the protein loading control. One representative blot of three independent experiments is shown. (**C**) Comparison of total citrullinated protein levels detected in mock- vs. HSV-2-infected HFFs using the Rh-PG probe. Values are expressed as means ± SEM of four independent experiments, * *p* < 0.05, **** *p* < 0.0001; one-way ANOVA followed by Bonferroni’s post-test.

To visualize proteins undergoing citrullination in complex proteomes, several phenylglyoxal (PG)-based probes have been developed [28]. Specifically, the rhodamine-PG (Rh-PG) compound covalently modifies the urea group of citrulline under acidic conditions, and the resulting adduct can be directly visualized by SDS-PAGE (Figure 1A). Thus, we first labeled citrullinated proteins in whole cellular protein extracts from HSV-2-infected HFFs with Rh-PG and then separated them on an SDS-PAGE gel. As shown in Figure 1B, starting from 16 h post infection (hpi), protein citrullination levels in HSV-2-infected HFFs were significantly increased compared to mock-infected controls, paralleled by the expression of the immediate early viral protein ICP8. Of note, and in line with our earlier findings with HSV-1 [21], we consistently found a distinct increase in bands corresponding to proteins with a molecular weight higher than 75 kDa. Finally, the total fluorescence intensities of the bands corresponding to citrullinated targets were quantified relative to the expression of β-actin, showing a significant increase starting from 24 hpi (Figure 1C).

**Figure 2:**
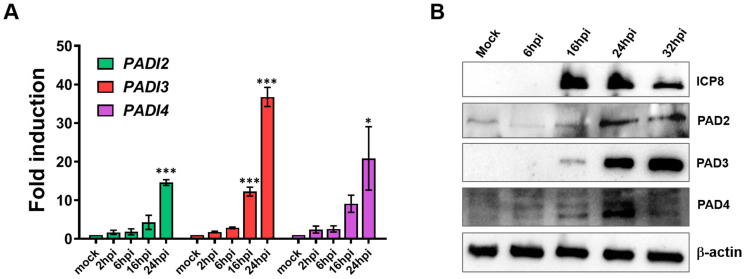
HSV-2 infection increases PADI3 expression in human fibroblasts. (**A**) mRNA expression levels of PADI isoforms by RT-qPCR of HSV-2-infected vs. uninfected (mock) HFFs, normalized to GAPDH and expressed as mean fold change ± SEM over mock-infected cells. * *p* < 0.05, *** *p* < 0.001; one-way ANOVA followed by Bonferroni’s post-test. (**B**) Protein lysates from uninfected (mock) or infected HFFs were analyzed by immunoblotting at different time points using the indicated antibodies. A representative blot from three independent experiments is presented.

Having previously identified *PADI3* as essential for HSV-1 infection despite its transcriptional induction being similar to those of *PADI2* and *PADI4* at 16 hpi in HSV-1-infected HFFs [21], we next sought to determine which PAD enzymes contribute to the altered citrullination profile during HSV-2 infection. RT-qPCR analysis of HSV-2-infected HFFs revealed that the expression levels of *PADI2*, *PADI3*, and *PADI4* genes were significantly upregulated in HSV-2-infected HFFs at 24 hpi compared to mock-infected controls, albeit to varying extents: 15-fold for *PADI2*, 37-fold for *PADI3*, and 21-fold for *PADI4* (Figure 2A). Of note, *PADI3* was uniquely upregulated by 12-fold compared to the mock-infected control as early as 16 hpi. Consistent with its mRNA expression, PAD3 protein levels were also increased upon HSV-2 infection (Figure 2B). In particular, the protein was detectable from 16 hpi onward, concurrent with the expression of the viral ICP8 protein, displaying a pattern similar to what we observed during HSV-1 infection [21]. Similar to observations made during HSV-1 infection, PAD2 and PAD4 protein levels were slightly increased in HSV-2-infected cells at 24 hpi. However, this upregulation was relatively modest when compared to that of PAD3 and was considerably reduced at 32 hpi (Figure 2B and Supplementary Figure S1).

Overall, these findings indicate that, akin to HCMV and HSV-1, HSV-2 can induce overall protein citrullination. Furthermore, this induction appears to be driven by the same PAD3-mediated mechanism observed during HSV-1 infection.

### 2.2. Evaluation of the Antiviral Activity of PAD Inhibitors

**Figure 3:**
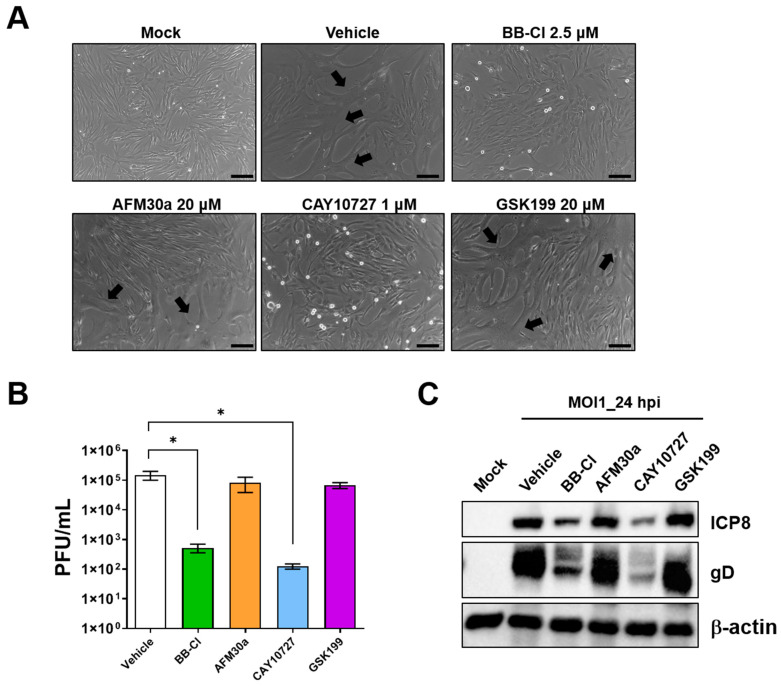
Targeting PAD3 hampers HSV-2 replication in human cells. (**A**) Representative images of infected HFFs (24 hpi) at an MOI of 1 PFU/cell treated with various PAD inhibitors or vehicle (DMSO) (syncytia formation = black arrows). Scale bars: 20 μm. (**B**) Plaque assay on supernatant of HSV-2-infected HFFs (MOI 1) collected at 24 hpi and treated with BB-Cl-amidine (2.5 μM), AFM30a (20 μM), CAY10727 (1 μM), GSK199 (20 μM), or vehicle. Values are shown as means ± SEM (error bars) from four independent experiments, * *p* < 0.05; non-parametric paired *t*-test. (**C**) Protein lysates from uninfected (mock) or infected HFFs (24 hpi) at an MOI of 1 PFU/cell treated as in (**B**) were analyzed by Western blot to assess viral expression with anti-ICP8 and anti-gD antibodies; β-actin was employed as a loading control for cellular protein expression. One representative blot of three independent experiments is shown.

Having established that HSV-2 positively impacts the expression levels of PAD2, 3, and 4, we next sought to determine whether suppressing the enzymatic activity of these proteins—especially that of PAD3—would influence viral replication. For this purpose, we treated HSV-2-infected HFFs with a panel of PAD inhibitors at non-toxic concentrations. This included the pan-PAD inhibitor BB-Cl-amidine (BB-Cl) and one PAD-specific inhibitor for each of the three isoforms, AFM30a for PAD2, CAY10727 for PAD3, and GSK199 for PAD4, as well as equal volumes of the vehicle control. The treatments started 1 h prior to HSV-2 infection (MOI 1) and were maintained throughout the infection. At 24 hpi, supernatants and total cellular protein extracts were collected separately. As shown in Figure 3A, only BB-Cl and the PAD3-specific inhibitor significantly prevented syncytia formation (black arrows), a classical HSV-2-related cytopathic effect, and the infected HFFs displayed a morphology similar to that of mock-infected cells. In contrast, cells treated with AFM30a or GSK199 displayed a morphology similar to vehicle-treated cells. Moreover, total virus production was quantified in the supernatants by a plaque assay, showing that BB-Cl and CAY10727 significantly decreased viral release by two to three logs (Figure 3B). Consistently, immunoblot analysis of the cellular protein performed to quantify viral protein expression revealed that both PAD2 and PAD4 inhibitors had minimal effects on HSV-2 replication, while exposure to BB-Cl and CAY10727 strongly inhibited the expression of both early (ICP8) and late (gD) viral proteins.

**Figure 4:**
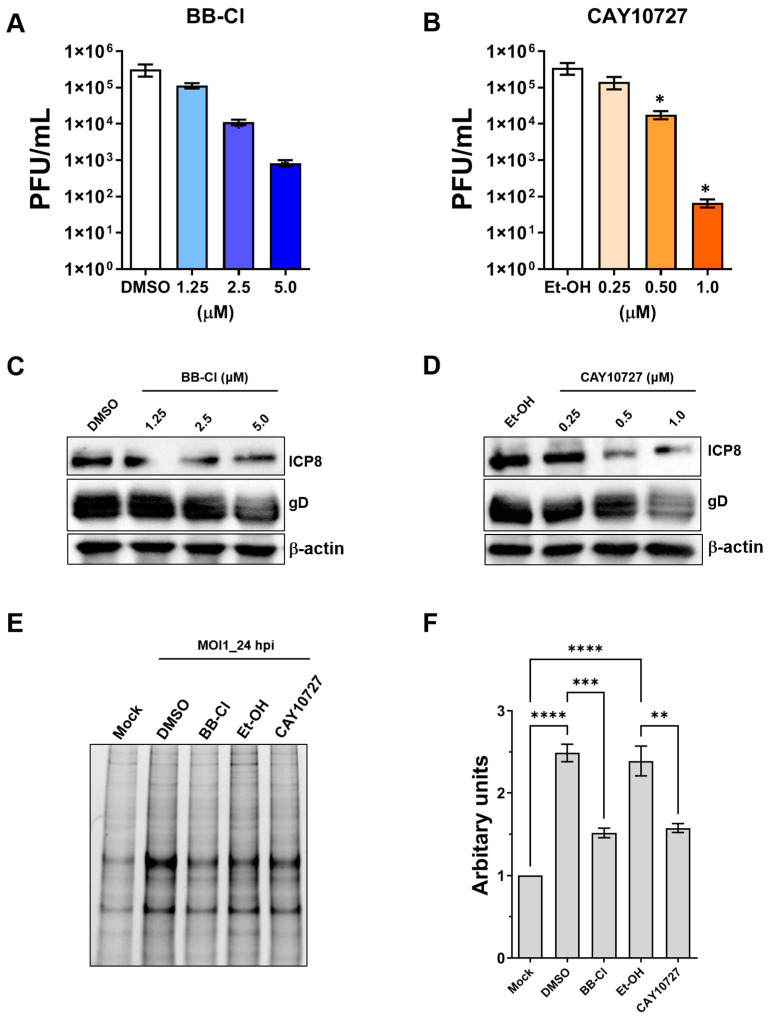
Antiviral activity and IC50 calculation of PAD inhibitors. HFFs were infected with HSV-2 (MOI 1 PFU/cell) and subsequently treated with the indicated concentrations of BB-Cl (**A**) or CAY10727 (**B**). Treatments were administered 1 h before virus adsorption and maintained throughout the experiment. At 24 hpi, viral plaques were counted under a microscope, and the number of plaques are plotted against the inhibitor concentration. Values are presented as means ± SEM (error bars) of four independent experiments. Values are expressed as means ± SEM (error bars) from four independent experiments, * *p* < 0.05; one-way ANOVA followed by Dunnett’s post-tests. (**C**,**D**) Protein lysates from uninfected (mock) or infected HFFs (24 hpi) at an MOI of 1 PFU/cell treated as in (**A**,**B**) were subjected to immunoblotting to assess viral expression using anti-ICP8 and anti-gD antibodies; β-actin served as a control for equal loading. One representative blot of three independent experiments is shown. (**E**) Protein lysates from uninfected (mock) or infected HFFs (24 hpi) at an MOI of 1 PFU/cell treated with CAY10727 (0.5 µM), BB-Cl (2.5 µM), or vehicle (DMSO or Et-OH) were labeled with the Rh-PG probe and subjected to gel electrophoresis to detect citrullinated proteins. (**F**) Comparison of total citrullinated protein levels detected in (**E**). Values are presented as means ± SEM of three independent experiments, * *p* < 0.05, ** *p* < 0.01, *** *p* < 0.001, **** *p* < 0.0001; one-way ANOVA followed by Bonferroni’s post-test.

We next determined the half-maximal inhibitory concentration (IC50) of the two compounds that displayed HSV-2-inhibitory activity. For this purpose, we treated HSV2-infected HFFs with increasing concentrations of BB-Cl and CAY10727 or with equivalent amounts of vehicle control (DMSO or Et-OH). We then assessed the total amount of HSV-2 production by a plaque assay after continuously exposing HFFs to the PAD inhibitors for 24 h. Both inhibitors showed a dose-dependent reduction in the quantity of viral particles (Figure 4A,B). Specifically, BB-Cl and CAY10727 had IC50 values of approximately 1 μM and 0.3 μM, respectively, which were very similar to the values previously observed for HSV-1. Congruently, viral protein synthesis also decreased in a dose-dependent manner (Figure 4C,D).

Finally, we employed the citrulline-specific probe Rh-PG to assess the total protein citrullination profile in HSV-2-infected HFFs treated with PAD inhibitors (Figure 4E). Quantification of the total fluorescence intensities of the bands corresponding to citrullinated targets (Figure 4F) confirmed that PAD inhibitors effectively reduce the overcitrullination triggered by HSV-2 infection.

Overall, these results indicate that PAD3 may be a promising target for the treatment of HSV-2-associated infections.

### 2.3. Genetic Depletion of PADI3 Affects HSV-2 Replication

**Figure 5:**
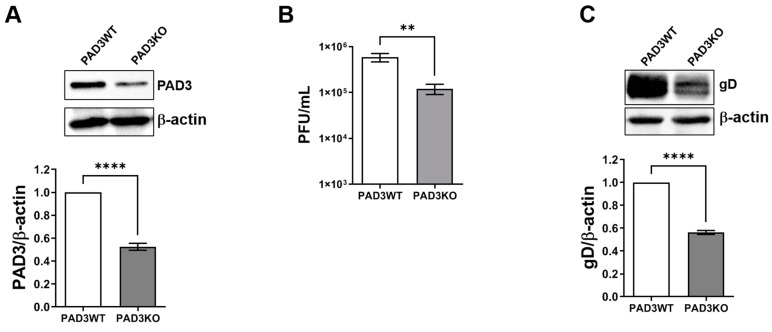
Genetic ablation of PADI3 gene impairs HSV-2 replication. PAD3 knockout (PAD3KO) HFFs were created using CRISPR/Cas9 technology. (**A**) The effectiveness of PAD3 protein depletion at 24 hpi was evaluated by Western blot with antibodies against PAD3 or β-actin, with the latter serving as the protein loading control. Relative densitometric analysis, representative of three independent experiments, presents values expressed as fold change in PAD3 expression normalized to β-actin. (**B**) PAD3KO HFFs were infected with HSV-2 (MOI 1). At 24 hpi, the viral supernatants were collected and quantified by the standard plaque assay. Values are expressed as mean ± SEM relative to three independent experiments, ** *p* < 0.01, **** *p* < 0.0001 non-parametric *t*-test. (**C**) Western blot analysis on protein lysates from PAD3WT (ctrl) or PAD3KO HFFs infected with HSV-2 (MOI 1) for 24 h. Viral protein expression was determined using the anti-gD antibody, with β-actin expression used to verify equal protein loading. Relative densitometric analysis, representative of three independent experiments, shows values expressed as fold change in viral gD expression normalized to β-actin.

To confirm the antiviral activity attributed to the specific inhibition of PAD3 by the compound CAY10727, rather than to an off-target effect, we generated PADI3 knockout (PAD3KO) HFFs using CRISPR/Cas9 technology with two different guide RNAs (gRNAs). Given the low detectability of PAD3 by Western blot in uninfected cells, we evaluated the silencing effect post-HSV-2 infection, which, according to our results shown in Figure 2, promotes PAD3 overexpression. Immunoblotting revealed a substantial reduction in PAD3 expression, with PAD3KO cells displaying about a 60% decrease compared to cells transfected with an empty vector (PAD3WT) (Figure 5A,B).

To assess the necessity of PAD3 for HSV-2 replication, we quantified total viral production using a plaque assay in both PAD3KO and PAD3WT HFFs infected with HSV-2 at an MOI of 1. At 24 hpi, we found that the viral load in PAD3KO cells was approximately an order of magnitude lower than that in control cells (Figure 5B). Furthermore, immunoblot analysis at 24 hpi showed a consistent decrease in the expression of the late viral protein gD in PAD3KO cells (Figure 5C).

Taken together, these findings indicate that PAD3 expression is necessary for optimal HSV-2 replication in HFFs.

## 3. Discussion

In the present study, we expand our research on the antiviral activity of PAD inhibitors against herpesviruses. Using specific probes for anti-citrullinated proteins and qPCR analyses, we show that HSV-2 infection modifies the overall citrullination profile in HFFs. This alteration results from the activation of the PAD3 isoform at both mRNA and protein levels, beginning at 16 hpi. Further supporting these findings, we find that the PAD3-specific inhibitor CAY10727, similar to the broader-spectrum PAD inhibitor BB-Cl, effectively counters the cytopathic effects typically induced by HSV-2 in HFFs. In addition, it reduces total virus release in the supernatants and total viral expression, showing an IC50 value similar to that previously observed for HSV-1 [21].

These results are reinforced by genetic experiments where the ablation of PADI3 in HFFs significantly impacts both total viral production, as measured by a plaque assay, and the expression of the late viral protein gD. This confirms a distinct mechanism by which different viruses post-translationally modify host and/or viral proteins to boost their replication. Notably, the mechanism used by alphaherpesviruses HSV-1 and HSV-2 [20] differs from what we observed with the betaherpesvirus HCMV, which strongly induces the PAD2 and PAD4 isoforms [20].

Viruses exploit host cellular machinery for replication and evolve rapidly, often leading to drug resistance. Targeting host factors crucial for viral life cycles offers a promising therapeutic approach, as it interferes with viral replication and reduces the likelihood of resistance [26]. This strategy, unlike conventional antivirals, aims to disrupt the viral manipulation of host processes, enhancing the effectiveness of treatments against viral infections [29]. In this regard, recent studies have highlighted the importance of citrullination, a PTM involved in numerous inflammatory-based diseases [13]. This has led to the development and commercial availability of an increasing number of PAD inhibitors targeting the enzymes that catalyze citrullination [30,31]. Thus, exploring the role of citrullination in viral infections, such as HSV-2, and the potential of PAD inhibitors as antiviral agents represents a novel and promising direction for therapeutic research.

In this scenario, identifying the specific PAD isoform(s) that viruses exploit to favor their replication is critical for the development of effective HATs. Indeed, the use of specific inhibitors that target a distinct isoform rather than all isoforms, as in the case of pan-PAD inhibitors, may significantly minimize off-target effects and toxicity while increasing the effectiveness of the antiviral treatment [30].

PAD3 is primarily found in the most differentiated layers of the epidermis and hair follicles, where structural proteins, such as filaggrin, are its main substrates [15]. Along with PAD1, the main function of PAD3 is to maintain the health of hair, toenails, and other tissues by regulating keratin levels. It is also highly expressed in human neural stem cells and modulates cell death throughout neural development in a calcium-dependent fashion. It is worth noting that it has not been detected in healthy human brains post-embryonic development [11].

This study provides compelling evidence that, similar to HSV-1, HSV-2 mainly alters the protein citrullination profile by transcriptionally activating *PADI3*. Even though *PADI2* and *PADI4* are also marginally increased at the mRNA levels, their overexpression does not match the scale observed during HCMV infection of HFFs, where *PADI3* expression remains basically unchanged [20].

Another important finding from our study is that the specific pharmacological ablation of PAD3 protein strongly inhibits HSV-2 replication, as demonstrated by the marked reduction in viral titers in the supernatants of infected cells and the reduced expression of viral early and late proteins in infected cells treated with safe doses of the drug.

Collectively, our findings reveal a central role for PAD3 protein in mediating a PTM strategy that HSV-2 exploits to foster its replication.

While our research does not directly link this phenotype with the modification of specific proteins involved in antiviral responses or restriction activity, given the very narrow PAD3 expression spectrum in human tissues and its high specificity of induction during HSV-1 and HSV-2 infections, it is tempting to speculate that PAD3-specific inhibitors may be suitable candidates for treating these infections. This aspect is particularly attractive as these inhibitors are expected to have a minimal impact on the physiological functions of the targeted enzyme while significantly curbing viral replication.

Currently, CAY10727 is the only commercially available PAD3-specific inhibitor that has not yet been tested in vivo. However, in recent years, citrullination has garnered significant interest in the scientific community, prompting the development of new and increasingly specific PAD inhibitors. Future studies on animal models are therefore essential to establish the pharmacokinetics and bioavailability of these inhibitors and to determine their therapeutic potential for various pathologies, including viral infections.

## 4. Materials and Methods

### 4.1. Cell Lines and Viruses

Human foreskin fibroblasts (HFFs; ATCC SCRC-1041), human HEK 293 from the human kidney (embryonic) (HEK 293T, ATCC^®^ CRL-3216™), and African green monkey kidney cells (Vero, ATCC CCL-81) were grown in Dulbecco’s modified Eagle’s medium (DMEM; Sigma-Aldrich, St. Louis, MI, USA) supplemented with 1% (*v*/*v*) penicillin/streptomycin solution (Euroclone, Pero, Italy) and heat-inactivated 10% (*v*/*v*) fetal bovine serum (FBS, Belize City, Belize) (Sigma-Aldrich). The HSV-2 clinical isolate was a generous gift from Valeria Ghisetti, Amedeo di Savoia Hospital, Turin, Italy, and was propagated in Vero cells and titrated by a standard plaque assay as described in Section 4.4.

### 4.2. Reagents and Treatments

The PAD inhibitors BB-Cl, GSK199, CAY10727, and AFM30a—also known as CAY10723—were obtained from Cayman Chemical. All these compounds were reconstituted in dimethyl sulfoxide (DMSO) or ethanol (Et-OH) according to their solubility. Immediately before use, the inhibitors were diluted to their final concentrations in the cell medium. For experiments involving PAD inhibitors, cells were pre-treated with the inhibitors for 1 h before being infected at an MOI of 1 by adding the virus directly to the existing medium. Two hours post-virus adsorption, the viral inoculum was removed, and fresh medium containing the same concentration of inhibitors was added. No further medium or inhibitors were added until the sample collection.

### 4.3. In Vitro Antiviral Assay

Human foreskin fibroblasts (HFFs) were cultured in a 24-well plate for 24 h and then treated as outlined in Section 4.2. Briefly, the cells were incubated with the specified PAD inhibitors at the indicated concentrations for 1 h, followed by infection with HSV-2 at an MOI of 1. The virus was directly added to the cell culture medium. After a 2 h virus adsorption period at 37 °C, the viral inoculum was removed, and the cultures were maintained in the medium with the specified treatments for 24 h. DMSO or ethanol served as negative controls. Cells and supernatants were then collected, pooled, and lysed through two freeze–thaw cycles. The extent of viral replication was measured by titrating the infectivity of the sample via a standard plaque assay on Vero cells.

### 4.4. Plaque Assay

Vero cells were seeded at a density of 3 × 104/well in a 96-well plate and inoculated 24 h later with 10-fold serial dilutions of the HSV-2 samples prepared as detailed in Section 4.3. After 48 h, the cells were fixed and stained with crystal violet solution, and plaques were counted under an optical microscope to determine the virus titer, which was calculated using the following formula: virus titer (PFU/mL) = number of plaques × 0.1 mL/dilution fold.

### 4.5. RNA Isolation and Quantitative Nucleic Acid Analysis

Total RNA was isolated using the NucleoSpin RNA kit (Macherey-Nagel, Düren, Germany). One microgram of RNA was reverse-transcribed using the Revert-Aid H-Minus FirstStrand cDNA Synthesis Kit (Thermo Fisher Scientific, Waltham, MA, USA), according to the manufacturer’s instructions. A comparison of mRNA expression between samples (i.e., infected vs. mock-infected) was performed using SYBR green-based RT-qPCR on a Biorad CFX96 apparatus with the following primers (5′-3′): GAPDH Fw AGTGGGTGTCGCTGTTGAAGT; GAPDH Rv AACGTGTCAGTGGTGGACCTG; PAD2 Fw ACCTCCTCAGCCTCCCC; PAD2 Rv CCTACCTCTGGACCGATGTC; PAD3 Fw GCGTCCCATAGACCTCAAAC; PAD3 Rv CAGAGAATCGTGCGTGTGTC; PAD4 Fw CCTGTGGATTTCTTCTTGGC; PAD4 Rv GGGCACCTTGACTCAGCTT.

### 4.6. Western Blot Analysis

HFF cell lysates were prepared with RIPA buffer, and the protein content was quantified using the Bradford method, followed by immunoblotting. The primary antibodies used included anti-PAD3 (Abcam, Cambridge, UK, ab50246), anti-β-actin (Sigma-Aldrich, A2066), anti-gD (Virusys, HA025-1, Milford, MA, USA), and anti-ICP8 (Virusys, P1113). The secondary antibodies employed were anti-rabbit/mouse IgG and anti-mouse IgG, both linked to horseradish peroxidase and species-specific (Amersham, Merck Life Science S.r.l., Darmstadt, Germany). Blots were visualized using a Biorad Chemidoc Imaging System (Hercules, CA, USA).

### 4.7. Detection of Citrullination with Rhodamine-Phenylglyoxal (Rh-PG)

Whole-cell protein extracts were obtained as previously described [4,28]. Equal protein amounts were diluted with 80% trichloroacetic acid for precipitation and incubated with Rh-PG at a final concentration of 0.1 mM for 30 min at 37 °C. The reaction was stopped with 100 mM L-citrulline for 30 min at 4 °C in the dark. Samples were then centrifuged at 21,100× *g* for 10 min, washed with ice-cold acetone, and resuspended in 2X SDS loading buffer for gel electrophoresis. Gels were visualized (excitation = 532 nm, emission = 580 nm) using a Biorad Chemidoc Imaging System, and total fluorescence was quantified as described by Thompson et al. [14].

### 4.8. CRISPR-Cas9 Vector Constructs

The CRISPR/Cas9 system was used to create specific gene knockouts in HFFs. For this purpose, we employed a lentiviral CRISPR/Cas9 vector encoding a codon-optimized nuclear-localized Cas9 gene fused to the puromycin resistance gene via a T2A ribosome-skipping sequence (RP-557). This vector is also equipped with a human U6 promoter driving the expression of a guide RNA (gRNA; PAD3 gRNA = gTTACCCATAAATGTCCACGA), which is a fusion of a gene-specific CRISPR RNA (crRNA) and the trans-activating crRNA (tracrRNA) with a terminator sequence. An empty vector without gRNA was used as the negative control (WT cell line). All constructs were checked by Sanger sequencing (Chromas 2.6.6) [20].

### 4.9. Lentivirus Production and Transduction of HFFs with Lentiviral CRISPR/Cas9

Recombinant lentiviruses were packaged in HEK 293T seeded at a density of 3 × 104 in a 96-well plate. After 24 h, HEK 293T cells were cotransfected with the 3rd Generation Packaging System Mix (courtesy of A. Follenzi, University of Eastern Piedmont, Novara, Italy) and the aforementioned vectors using Lipofectamine 3000 (Thermo Fisher Scientific, Waltham, MA, USA) following the manufacturer’s instructions. Viral supernatants (9 mL) were collected after 72 h and centrifuged at 3000× *g* for 10 min at 4 °C to remove cellular debris. Recombinant lentiviral particles (3 mL) were used to transduce HFFs—seeded at a density of 2 × 105 in a 6-well plate—in the presence of 8 μg/mL of polybrene. Transduced cells were selected with puromycin (1 μg/mL) for 14 days post transduction. Successful gene knockout was subsequently confirmed via immunoblotting.

### 4.10. Statistical Analysis

All statistical analyses were performed using GraphPad Prism version 9.5.1 for Windows (GraphPad Software, San Diego, CA, USA). Data are shown as the means ± standard error of mean (SEM). Statistical significance was determined by the unpaired *t*-test (two-tailed) or one-way ANOVA with Bonferroni’s or Dunnett’s post-tests. A *p*-value of <0.05 was considered statistically significant, with significance levels denoted as follows: * *p* < 0.05; ** *p* < 0.01; *** *p* < 0.001. The half-maximal inhibitory concentration (IC50) was calculated using the Quest Graph IC50 Calculator (AAT Bioquest, Inc., https://www.aatbio.com/tools/ic50-calculator, accessed on 25 March 2024).

## Figures and Tables

**Table 1:** Peptidylarginine deiminase expression profiles, isoform-specific substrates, and disease involvement.

	Tissue Distribution	Citrullination Substrate	Biological Process	Disease or Role in Disease/Molecular Target (If Applicable)
**PAD1**	Uterus, kidney, proximal digestive tract, testis, bone marrow, prostate, placenta, lung, liver, muscle, spleen, pancreas, thymus, colon [6,11].	Keratin1, keratin 10 and filaggrin [11].	Skin differentiation, terminal differentiation of keratinocytes, epithelial-mesenchymal transition [11,12].	Psoriasis (changes in skin differentiation pathways/keratin) [13].
**PAD2**	Blood, brain, bone marrow, breast, eye, lung, proximal digestive tract, gastro-intestinal tract, testis, placenta, rectum, colon, salivary gland, gall bladder, spleen, epidermis, pancreas, spinal cord, pituitary gland, inner ear, kidney, olfactory tissue, thymus, neutrophils, macrophage, monocytes [6,11].	MBP, vimentin, GFAP, fibrinogen, histone H3, enolase 1, enolase 2, aldolase 1, aldolase 3, malate dehydrogenase 1, voltage-dependent anion channel 1, peroxiredoxin 1, cofilin 1, peptidylprolyl isomerase a, heat shock protein 8, MMP-9, actin, GRP-78, CXCL8, CXCL10, TNF-α [11].	Oligodendrocyte differentiation and myelination, brain plasticity, female reproduction, transcription regulation [11,12].	Multiple sclerosis, rheumatoid arthritis, Alzheimer disease, prion disease, COVID-19 [13,14].
**PAD3**	Hair follicles, neural stem cells, urinary bladder, muscle proximal digestive tract, prostate, vagina, epidermis, thymus, mammary gland, neutrophils [6,11].	Trichohyalin, S100A3, filaggrin, apoptosis-inducing factor, vimentin, tubulin, histone H2 [11].	Skin differentiation, hair follicle formation, terminal differentiation of keratinocytes [11,12].	Glioblastoma multiforme, psoriasis, bullous congenital ichthyosiform erythroderma, and central centrifugal cicatricial alopecia [13,15].
**PAD4**	Blood, lung, bone marrow, spleen, brain fat, vagina, placenta, eye, neutrophils, monocytes, macrophages, eosinophils [6,11].	ADAMTS13, histone H3, ING4, p300, nucleophosmin, nuclear lamin c, GSK3b, NF-kB-p65, collagen type I, collagen type II [11].	Chromatin decondensation, transcription regulation, tumor formation, innate immune response and NETosis process [11,12].	Rheumatoid arthritis, multiple sclerosis, cancers, COVID-19 [7,9,13,14].
**PAD6**	Blood, muscle, bone marrow, testis, spleen, small intestine, lung, liver, ovary, thymus [6,11].	α-tubulin [11].	Cytoskeletal reorganization in the egg and early embryo, preimplantation cleavage, early embryonic development, oocyte cytoskeletal sheet formation and female fertility, and target for contraceptive drugs [11,12].	Infertility [13].

## Data Availability

Data is contained within the article and Supplementary Materials.

## Supplementary Materials

The following supporting information can be downloaded at https://www.mdpi.com/article/10.3390/ijms25168709/s1.
