# Supplementary material for: Targeting Peptidylarginine Deiminase 3 to Efficiently Suppress Herpes Simplex Virus Type 2 Infection
Source: Int J Mol Sci. 2024 Aug 9;25(16):8709. doi: 10.3390/ijms25168709 (PMC11354815; doi:10.3390/ijms25168709)
Supplement: Supplementary file 1 [file ijms-25-08709-s001.zip › ijms-3059668-supplementary.pdf]

Supplementary Figure

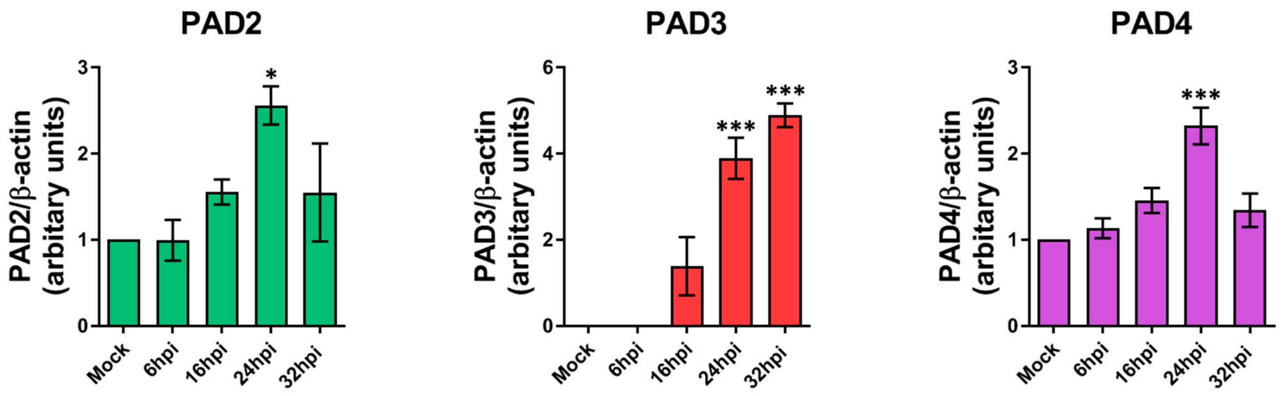

Supplementary Figure S 1. Densitometric analysis of three independent Western blots (one representative is shown in Figure 2B). Values are expressed as fold change in PAD2, PAD3 and PAD4 expression normalized to β-actin expression. Values are expressed as means ± SEM of four independent experiments, * $p < 0.05$, *** $p < 0.001$; one-way ANOVA followed by Bonferroni's post-test.
